# Implementation of problem-based learning modules in an introduction to public health course

**DOI:** 10.3389/fpubh.2024.1405227

**Published:** 2024-06-28

**Authors:** Yui Matsuda, Ashley Falcon, Andrew Porter, Aaron Royer, Leah Mohnkern, Diana Vergara, Yesenia Valiente

**Affiliations:** ^1^School of Nursing and Health Studies, University of Miami, Coral Gables, FL, United States; ^2^Platform for Excellence in Teaching and Learning, University of Miami, Coral Gables, FL, United States; ^3^Office of University Accreditation, University of Miami, Coral Gables, FL, United States

**Keywords:** problem-based learning, public health, undergraduate, active learning, education, students

## Abstract

**Introduction:**

With traditional lecture-based learning methods often criticized for their limited ability to foster critical thinking and cognitive engagement, problem-based learning (PBL) has emerged as a promising alternative. This research investigates the impact of PBL on student learning outcomes, specifically focusing on the development of higher-order thinking skills, communication, growth mindset, and metacognitive abilities.

**Methods:**

PBL was implemented in an undergraduate public health course at a private university in the southeast US. The study was conducted in the Spring of 2022 using a convergent mixed-methods approach. Quantitative data were derived from university-wide Quality Enhancement Plan surveys and a course-specific PBL survey, which were analyzed using Repeated Measures ANOVA to assess changes in student perceptions over time. Additionally, qualitative data from open-ended survey questions were analyzed through thematic analysis, providing deeper insights into the students’ experiences and perceptions of PBL.

**Results:**

Results indicated significant improvements in student communication skills, growth mindset, and metacognitive abilities across the semester. The thematic analysis of qualitative responses corroborated these findings, revealing enhanced team collaboration, active engagement in problem-solving, and increased comfort with complex real-world issues.

**Discussion:**

The findings contribute to the growing body of evidence supporting PBL and offer practical insights for implementing PBL in public health education. The study also highlights the need for institutional support in adopting innovative teaching methods like PBL, emphasizing faculty development, resource allocation, and curriculum design.

## 1 Introduction

Higher education institutions have long relied on lecture-based learning in courses across disciplines. Lectures expose students to new content and concepts so they can apply them in problem sets or other activities afterward; however, in their more passive forms, they come with a number of well-documented drawbacks, including failure to promote critical thinking and cognitive engagement (1). Active learning, an umbrella term referring to a range of instructional methods and activities, has become an increasingly popular alternative and aims to remedy these issues by shifting the focus from information transmission to active engagement with content, which crucially requires higher-order thinking and metacognition (2). Active learning has many benefits, including improved information retention and transfer, increased motivation, enhanced teamwork and communication skills, and lower course failure rates (3).

One form of active learning that has become increasingly recognized in recent years is problem-based learning (PBL), a method in which students learn new concepts as they work in small groups to solve open-ended, real-world problems with guidance but little knowledge transfer from the instructor. In PBL, students employ and develop their higher-order thinking skills “while eliciting information from personal real-life experiences and acquiring determinate knowledge about their own learning” (4). The PBL cycle, which is used to facilitate student learning (5), starts with students identifying what they know about the given problem. Based on the problem and the end goal, students organize their thoughts and identify what they need to know. Then, students will search, find, and engage with the needed information. After that, they will apply the new knowledge to the problem and ultimately find solutions toward the given problem. Students will work with their team members in this process. The primacy of context and content application in PBL is a notable departure from more traditional methods such as the lecture, which starts with content and moves to applications and one that more closely mirrors how people learn in the workplace. Furthermore, in line with active learning, there is ample and growing evidence for the effectiveness and benefits of PBL. Jimenez-Mejias et al., for example, found a strong causal relationship between problem-based learning, increased student satisfaction, and increased student performance, as did Li et al., who observed that students reported higher levels of personal satisfaction and motivation when participating in PBL (6, 7). In addition to observable higher student achievement, students report greater development of reasoning, independent thinking, and teamwork skills in PBL coursework versus traditional learning (8). Students also report that PBL builds on transferable skills that can be applied to their future workplace (9).

Despite the preceding evidence, the adoption of PBL at higher education institutions across the globe remains relatively low. Within this limited literature, the majority of the studies are related to PBL for postgraduate STEM programs such as medical and pharmacy schools (10). Furthermore, the research involving PBL instruction in undergraduate coursework is even more limited, particularly in public health education. The few studies on PBL in public health education predominantly center around student satisfaction with PBL (11), with a small number of others focusing on self-assessment, peer assessment, group dynamics, or the benefit of technology in teaching such courses (11, 12). To better understand the impact of PBL on undergraduate students taking a public health course, additional research is needed. Therefore, this study aims to evaluate the implementation of PBL in the Introduction to Public Health course at a university in the southeast US. Specifically, the authors quantitatively and quantitatively explored changes in student learning outcomes over the course of the semester to understand students’ learning via PBL.

The Introduction to Public Health course is offered through the Faculty Learning Community Fellowship, which unites faculty across the university for interdisciplinary exploration and discussion on the integration of innovative, discussion-oriented methods such as PBL. This initiative is part of the University’s Quality Enhancement Plan, the theme of which is *Learning through Dialogue and Discussion*. After the principal investigator completed the Faculty Learning Community Fellowship in 2021, Introduction to Public Health was the first official course at our university to fully implement PBL. The lack of available research supporting PBL in undergraduate public health education and the position of the Introduction to Public Health course as a trailblazer in PBL at our university are factors that create the optimal context for this study.

## 2 Materials and methods

The PBL modules were implemented in an undergraduate, in-person introductory public health course offered in spring 2022. This course broadly covers basic public health concepts and major issues in public health. There were three separate PBL problem sets: “Gentrification in Wynwood,” “COVID-19 reopening plan for a senior center,” and “Addressing college student health.” Before the start of each problem set, a related lecture was delivered in class. For the gentrification problem set, a lecture on environmental health was delivered. Lectures on communicable diseases and vaccines were provided for the COVID-19 reopening plan problem set. For the college student health problem set, two guest lecturers from the university’s student health and counseling center were invited to speak about the health issues among college students. Each problem set was allocated three class periods of 1 h each to examine the issues using a worksheet developed by the instructor in consultation with the PBL mentor at the university. For example, for the gentrification problem set, students examined the impacts of gentrification on long-term residents during the first class; they explored if and how gentrification positively influences the neighborhood during the second class and used the final class period to explore potential solutions to recommend to government officials. Their deliverable at the conclusion of this problem set was a group infographic to summarize what they addressed during each class period, including proposing potential solutions to the mayor from the perspectives of public health professionals. Students worked on the PBL problem set in groups: Groups of 4–5 were formed by students, who then worked with the same team members throughout the semester. A pre-class assignment was completed by each student, involving reading/watching 2–3 assigned sources to better understand the issues. In addition, each student was required to identify and familiarize themselves with at least one additional source of information, whether in the form of peer-reviewed or non-peer-reviewed articles, podcasts, or videos. In class, students discuss and work together on the issue at hand based on the pre-class assignments by identifying what they already know and need to know to understand the issue better and work toward solving the problems.

Data obtained for this study include the university-wide Quality Enhancement Plan surveys and a course-specific PBL survey. The university-wide Quality Enhancement Plan survey questions were developed by the staff members from the Office of University Accreditation who oversee university-wide assessments at the authors’ university. The survey was administered and managed by the Office of University Accreditation. Students are asked to complete self-assessment surveys wherein they rate their mastery of the Quality Enhancement Plan student learning outcomes in three areas: communication, growth mindset, and metacognition. Communication items ask respondents about their ability to communicate and engage in dialogue while respecting others’ opinions and viewpoints. Growth mindset items are asking about respondents’ attitudes as a learner. Metacognition items are about respondents’ ability to analyze the information and own it to create potential solutions. These surveys have been distributed in different periods throughout the semester. The first survey, Student Self-Assessment I (pre-survey), was sent out before the start of the term. The second survey, Student Self-Assessment II (Mid-point Survey), was sent around the middle of the term, and the third survey, Student Self-Assessment III (post-survey), was sent at the end of the semester. A course-specific PBL survey was created by the instructor and administered on the last day of class to quantitatively and qualitatively assess students’ general satisfaction and perceptions of the overall PBL experience. Open-ended questions probed what students believed was the most important thing they learned during the PBL, structure of PBL they found helpful, and any improvements to be made for future PBLs. All surveys were administered electronically: The university-wide surveys were mounted on Course Evaluations from Anthology, Inc., and the course-specific survey was administered using Qualtrics (13). Inclusion criteria consisted of enrollment in the introductory public health course in the spring of 2022 and willingness to complete the anonymous surveys. Students not enrolled in the course or enrollees not willing to complete the surveys were excluded. The survey was anonymous and voluntary to reduce potential sources of bias. The university’s institutional review board approval was obtained prior to the data collection, and the study consent form was read by students, who then agreed to complete the survey prior to data collection.

## 3 Data analysis plan

In this study, we used both quantitative and qualitative data analyses to understand the impact of PBL on students in a public health course.

Quantitative data collected from student surveys were converted from long format to wide format. These data were analyzed to determine if there were any notable changes in students’ skills and attitudes over the course of the semester. To analyze these changes, the authors employed Repeated Measures ANOVA. This statistical technique determines if the differences in survey responses are significant, that is, if students’ experiences in PBL had a meaningful impact on their learning and development. For comparisons based on demographic characteristics, a variable was calculated for each item to indicate growth on that item. Additionally, the authors examined students’ responses based on their demographic characteristics, using one-way ANOVA and independent samples *t*-tests, which help us understand if different groups of students experience the course in different ways. To allow for the use of demographic variables in analyses, string variables were recoded into numeric variables with variable labels. SPSS version 27 was used to carry out analyses.

Qualitative data obtained from open-ended survey questions were evaluated using a thematic analysis approach (14). This process allowed us to gain deeper insight into how students feel about their PBL experience and to understand the nuances of their learning journey. Two reviewers independently identified themes within and across qualitative responses and then discussed findings until consensus was achieved. Quotes that exemplify central themes were selected to demonstrate student input with fidelity. Then, a convergent mixed methods design was used to integrate quantitative findings with qualitative findings (15). In a convergent mixed methods design, researchers collect quantitative and qualitative data concurrently and bring the findings together so they can be examined together.

## 4 Results

Participant demographics and repeated measures ANOVA analyses for all survey items are provided in Tables 1, 2 below. Sixty-six students completed the study. Survey completion rates were 87.14% (pre-survey), 70.59% (mid-point survey), and 64.71% (post-survey). The majority of students identified themselves as female, and the participants came from a variety of races/ethnicity. The majority of students came from health-related majors (e.g., health sciences, nursing).

**Table 1:** Frequencies for participant demographic and background information (*n* = 66).

	*n*	%
**Gender**		
Female	49	74.2
Male	17	25.8
**Race/ethnicity**		
American Indian/Alaskan Native	1	1.5
Asian	6	9.1
Black	5	7.6
Hispanic	22	33.3
White	28	42.4
Multiple specified	3	4.5
None specified	1	1.5
**Major**		
Bachelor of Art	9	13.6
Bachelor of Science	21	31.8
Bachelor of Health Science/Public Health	26	39.3
Bachelor of Science in Nursing	6	9.1
Other (e.g., business administration, industrial engineering)	2	3
Unknown	2	3

**Table 2:** Repeated measures ANOVA results for overall sample.

Item	*F*	$\eta_{p}^{2}$	p -value
Comm1: I present ideas clearly	13.71	0.28	<0.001
Comm2: I transition smoothly from topic to topic	37.43	0.52	<0.001
Comm3: I respect points of view that differ from my own	6.18	0.15	0.02
Comm4: I find value in discussing particular topics or problems	2.26	0.06	0.14
Comm5: I encourage others to talk about relevant topics or problems	16.76	0.32	<0.001
GroMind1: I take charge of my own learning	5.81	0.14	0.02
GroMind2: I learn from my mistakes	0.02	0.001	0.89
GroMind3: I accept feedback from fellow classmates in discussion and problem-solving	11.51	0.25	0.002
MetaCog1: I summarize or identify course materials of assignments when necessary	4.57	0.12	0.04
MetaCog2: I summarize new themes or concepts from course materials or assignments	9.07	0.21	0.005
MetaCog3: I connect or integrate ideas from previous class sessions	7.68	0.18	0.009
MetaCog4: I refer to patterns in discussions or when solving a problem	4.10	0.11	0.05
MetaCog5: I use multiple strategies to analyze information or a problem	10.11	0.22	0.003
MetaCog6: I examine an old topic or problem with new ideas/perspectives	4.21	0.11	0.05
MetaCog7: I talk about pros and cons when discussing a topic or solving a problem	3.03	0.08	0.09
MetaCog8: I take into account the complexities of a topic or problem when I justify my position or use an approach	13.31	0.28	<0.001

$\eta_{p}^{2}$ < 0.06, small effect; < 0.14, medium effect; ≥ 0.14, large effect.

### 4.1 Overall trends

Overall trends for each survey item evaluated through repeated measures ANOVA suggest that most measures for student perceptions regarding PBL experiences showed significant changes across the three timepoints. Results are organized based on the subscale below. A complete summary of ANOVA results is provided in Table 2. Themes identified from the qualitative analysis and representative quotes are listed in Table 3.

**Table 3:** Qualitative themes and sample quotes.

Themes	Quotes
**Improved communication**	
Idea sharing	“[PBL] has helped me communicate my ideas more clearly and focused with my group”
Effective listening	“We did not have any talking over each other, and we were all listening to each other and let ideas of everyone be expressed”
Comfort with collaboration	“We have all gotten more comfortable with each other and I found that our conversation has significantly gotten better”
Open-mindedness and respect for differing opinions	“[PBL] helped me learn to fully listen to others’ opinions and ideas as well as integrate them into my ideas and build them even more”
Team-based communication skill development	“I would say one of the most important things I learned through the PBL process was how to work with a team, it differs from the [service-learning project] because we were actually engaging in a zoom together or many class periods in a row, and each member of our team did a good job at listening to different thoughts and opinions of others. I believe I feel stronger working in a team setting”
**Growth mindset**	
Coming prepare for PBL	“We all come to class prepared with resourceful information that helps navigate the class worksheet assigned”
Self-led efforts to promote learning	“It was having to look at the situation and problem as a whole from an un-biased viewpoint. This is difficult to do, but [PBL] really forces you to get all of the facts and improve your listening skills so that you can ultimately turn in the most cohesive solution”
Acceptance of peer input	“We all listened to each other’s ideas more and compromised”	Acceptance of peer feedback	“For the first PBL I had trouble with my group. I was taking on most of the responsibility. However, for the second PBL, we worked more as a group”
Enjoyment with applied learning	“I loved working with a group on a real-life problem. It was fun and interesting, and my group worked very well together”
**Metacognitive skills**	
Synthesizing and building on knowledge	“I think I got better at the process of information when reading resources with diverging viewpoints. It is very easy to pick sides and a lot harder to keep an open mind, which this project helped me a lot on. Also, working with my team was a great experience when we all put our heads together and expanded on each other’s thoughts and ideas, the work flowed very smooth”
Accounting for the complexity of a topic	“I learned about how gentrification can be super good for some, but really harmful to others. This is an example of the pros and cons of anything in life and made me aware of the critical thinking necessary to determine solutions”

PBL, problem-based learning.

### 4.2 Subscale 1: communication

#### 4.2.1 Quantitative results

Participants reported stronger agreement with the statement “I present ideas clearly” at later timepoints, compared with baseline levels (*p* < 0.001). Pairwise comparisons using Bonferroni adjustment indicate that there is a significant difference between baseline (*M* = 5.47, SE = 0.21) and timepoint 3 (*M* = 6.28, SE = 0.14), MD = 0.81, *p* = 0.002, but that no other pairwise comparisons were significant. Results for the second item in the communication practices subscale suggest that students had stronger agreement with the statement “I transition smoothly from topic to topic” at later timepoints, compared with baseline levels (*p* < 0.001). Pairwise comparisons using Bonferroni adjustment indicate that there is a significant difference between baseline (*M* = 5.11, SE = 0.18) and timepoint 2 (*M* = 5.88, SE = 0.19), MD = 0.778, *p* = 0.002, and between baseline and timepoint 3 (*M* = 6.22, SE = 0.15), MD = 1.11, *p* < 0.001.

The third communication practices item, “I respect points of view that differ from my own”, had stronger agreement at later timepoints compared with baseline (*p* = 0.02) but no significant pairwise comparisons. This item showed a slight decrease in agreement after the second time point, indicating a diminishing effect. The fourth item followed a similar pattern (*p* = 0.14), with no changes between timepoints 2 and 3.

The fifth item in the communication practices subscale mirrored the patterns of the first two (*p* < 0.001). Pairwise comparisons using Bonferroni adjustment indicate that there is a significant difference between baseline (*M* = 5.92, SE = 0.21) and timepoint 3 (*M* = 6.64, SE = 0.11), MD = 0.722, *p* < 0.001.

#### 4.2.2 Qualitative results

Communication was a recurring theme across open-ended, qualitative responses (Table 3). Several students acknowledged an improved ability to communicate ideas and listen effectively. One student noted, “[PBL] has helped me communicate my ideas more clearly and focused with my group.” In many instances, students identified that increased comfort with group members over time facilitated this improvement. Students also conveyed that they were able to remain open-minded and respectful of differing opinions, which facilitated better rapport, collaboration, and teamwork. A majority of students identified communication skills as one of the most important things they learned through their PBL experience, as exemplified by this one student, “I would say one of the most important things I learned through the PBL process was how to work with a team, it differs from the [service-learning project] because we were actually engaging in a zoom together or many class periods in a row, and each member of our team did a good job at listening to different thoughts and opinions of others. I believe I feel stronger working in a team setting.”

### 4.3 Subscale 2: growth mindset

#### 4.3.1 Quantitative results

The first two items of this subscale show growth from timepoint 1 to timepoint 2 but diminishing effects from timepoint 2 to timepoint 3. For the first item in this subscale, “I take charge of my own learning,” there is a significant overall effect (*p* = 0.02). Pairwise comparisons using Bonferroni adjustment indicate that there is a significant difference between baseline (*M* = 5.94, SE = 0.20) and timepoint 2 (*M* = 6.56, SE = 0.12), MD = 0.611, *p* = 0.009, but that no other pairwise comparisons were significant. For the second item, “I learn from my mistakes,” the overall effect is not significant.

The last item of this subscale shows a large increase in positive endorsement from timepoint 1 to timepoint 2 and a small increase thereafter (*p* = 0.002). Pairwise comparisons using Bonferroni adjustment indicate that there is a significant difference between baseline (*M* = 6.17, SE = 0.15) and timepoint 3 (*M* = 6.56, SE = 0.12), MD = 0.389, *p* = 0.005, but that no other pairwise comparisons were significant.

#### 4.3.2 Qualitative results

Qualitative data provided several instances where students exhibited initiative toward their learning (Table 3). Specifically, many students identified the importance of coming to class prepared to effectively engage in discussion and work with their group members. Students reported that they identified how best to promote their learning, ranging from comments specifying the need to overcome shyness and speak more to comments recognizing the need to talk less and listen more. One student shared, “It was having to look at the situation and problem as a whole from an un-biased viewpoint. This is difficult to do, but [PBL] really forces you to get all of the facts and improve your listening skills so that you can ultimately turn in the most cohesive solution.” Taking charge of their learning also included students’ acceptance of input from others. Many students reported that it was important to listen to and incorporate opinions and additional information from their peers. Likewise, several students also acknowledged receiving constructive feedback about imbalances in group member workload that they accepted and worked to resolve. Additionally, several students expressed enjoyment with the experience and all that they learned, with many citing the application of their knowledge as one of the most important things learned.

### 4.4 Subscale 3: metacognitive skills

#### 4.4.1 Quantitative results

All items in this scale follow similar patterns to the previous two subscales, with positive endorsement increasing more from timepoint 1 to timepoint 2 and either a slight increase or a decrease in endorsement between timepoint 2 and timepoint 3. Based on *post hoc* analyses, pairwise comparisons for MetaCog1 using Bonferroni adjustment indicate that there is a significant difference between baseline (*M* = 5.89, SE = 0.19) and timepoint 2 (*M* = 6.39, SE = 0.13), MD = 0.50, *p* = 0.03, but that no other pairwise comparisons were significant. Results for MetaCog2 (*p* = 0.04), MetaCog2 (*p* = 0.005), MetaCog4 (*p* = 0.05), and MetaCog7 (*p* = 0.09) all show significant changes over time, but diminishing effects between timepoints 2 and 3. For MetaCog1, pairwise comparisons using Bonferroni adjustment indicate that there is a significant difference between baseline (*M* = 5.69, SE = 0.15) and timepoint 2 (*M* = 6.33, SE = 0.14), MD = 0.639, *p* = 0.002, and baseline and timepoint 3 (*M* = 6.25, SE = 0.16), MD = 0.556, *p* = 0.01. *Post hoc* analyses for MetaCog4 followed the same pattern as MetaCog1, indicating that there is a significant difference between baseline (*M* = 5.69, SE = 0.20) and timepoint 2 (*M* = 6.29, SE = 0.16), MD = 0.600, *p* = 0.03. For MetaCog7, no pairwise comparisons were significant.

The remaining metacognitive skills items showed large increases from timepoint 1 to timepoint 2 and smaller increases from timepoint 2 to timepoint 3. *Post hoc* analysis of MetaCog3 (*p* = 0.009) suggests that there is a significant difference between baseline (*M* = 5.72, SE = 0.17) and timepoint 3 (*M* = 6.22, SE = 0.15), MD = 0.500, *p* = 0.03, but no other pairwise comparisons were significant. For MetaCog5 (*p* = 0.003), pairwise comparisons using Bonferroni adjustment indicate that there is a significant difference between baseline (*M* = 5.69, SE = 0.17) and timepoint 3 (*M* = 6.31, SE = 0.15), MD = 0.611, *p* = 0.009, but no other pairwise comparisons were significant. MetaCog6 (*p* = 0.05) did not result in any significant pairwise comparisons. MetaCog8 (*p* < 0.001) followed the same pairwise results pattern as MetaCog5, with significant differences between baseline (*M* = 5.64, SE = 0.17) and timepoint 2 (*M* = 6.31, SE = 0.14), MD = 0.667, *p* = 0.003, and baseline and timepoint 3 (*M* = 6.33, SE = 0.12), MD = 0.694, *p* = 0.003.

#### 4.4.2 Qualitative results

Students also provided open-ended qualitative responses demonstrative of metacognition (Table 3). Many students acknowledged embracing and piggybacking off others’ thoughts and working to synthesize their knowledge with that of their group members in an effort to have a more fully developed understanding and solution-oriented approach. Building on previous themes, one student noted, “I think I got better at the process of information when reading resources with diverging viewpoints. It is very easy to pick sides and a lot harder to keep an open mind, which this project helped me a lot on. Also, working with my team was a great experience when we all put our heads together and expanded on each other’s thoughts and ideas, the work flowed very smooth.” Some students further elaborated on this concept by identifying the importance of considering the complexity of a topic, including the varied perspectives and needs of different stakeholder groups.

### 4.5 Demographic comparisons

Changes in mean response for all items based on demographic characteristics were evaluated with independent samples t-tests or one-way ANOVA. The only significant difference found for the communication practices subscale was for Comm4. Results from a one-way ANOVA suggest that there is a significant difference in changes for this item ( $\eta_{p}^{2}$ = 0.31). *Post hoc* analysis could only be done after the removal of groups with less than two individuals. *Post hoc* analysis using Tukey adjustment indicates that Asian students had a significantly greater change in their agreement with this item compared to Black (*p* = 0.009), Hispanic (*p* = 0.002), and White students (*p* = 0.003). There were no other significant demographic differences in the change in response to any items.

The second subscale also had very few significant differences based on demographic characteristics. Results for GroMind1 show that participants who identified as male had a greater change in this item (*M* = 1.33, *n* = 9) compared with those who identified as female (*M* = 0.35, *n* = 34), *t*(41) = 2.01, *p* = 0.05, and the magnitude of the effect was moderate (Cohen’s d = 0.75). No other significant group differences were identified. No significant group differences were found for any items in the third subscale.

## 5 Discussion

This study evaluated students’ experience of problem-based learning in an undergraduate introductory public health course at a private university in the southeastern U.S. Overall, our quantitative and qualitative results are congruent and complemented each other. Regarding communication, a majority of quantitative items showed statistically significant increases from time 1 to 3, most with large effect sizes suggesting meaningful practical importance. Other studies have also found that their students’ communication skills have improved (16, 17). For our course, the instructor deliberately had students work with the same group throughout the semester, which seemed to positively contribute to students feeling comfortable when communicating with their group members. The qualitative data from this study also supports this notion of feeling comfortable communicating with the team members as the semester progressed. Comfort with group members allowed students to have more robust and transparent ‘dialogue’ where they could share ideas and safely express differences of opinion. This dynamic appeared to support more productive team-oriented collaboration.

In the growth mindset category, the increase between time 1 and 2 was greater than between time 2 and 3. The relative greater increase between times 1 and 2 is likely due to the learning and adjustment that occurred early on during the PBL process. As students started their PBL module, they prepared for and engaged with the problems and related discussions with peers unique to this way of learning. Findings from qualitative analysis nicely illustrated how students have enjoyed, learned from, and grown during this experience. The real-world nature of the PBL modules in particular was a feature that effectively interested and intrinsically motivated students to engage with course material and peers. The desire to find meaningful solutions to the presented problems appeared to stimulate students’ actions, including active knowledge acquisition from various sources and adapting how they work with their peers in an effort to improve both process and outcome. Other qualitative studies also found that PBL challenged how students learn and helped cultivate their growth mindset through the courses (18, 19).

In the metacognitive category, the overall trend is that metacognition improved. Some items had a larger increase in scores between times 1 and 2, potentially due to similar learning curves that occurred in the beginning, as discussed above regarding the growth mindset category (20, 21). The qualitative results illustrated that students learned to be able to work with their peers to solve complex problems with multiple layers of influence. Students conveyed an increased awareness and appreciation for seeking out and using various sources of information, including those shared by their peers. This intentional process of acquiring knowledge supported students’ efforts to generate solutions that were responsive to the nuance and intricacies of the topics explored.

Regarding the demographic comparison, it is notable that Asian students had a greater increase in agreement with the communication item about finding value in discussing particular topics or problems. While, as a group, Asians are incredibly heterogeneous and diverse, in general, Asians tend to value harmony, and their education in their respective countries tends to be done by teachers in an authoritative manner. Thus, discussing certain topics may not have been part of what they were accustomed to and valued before their PBL experience. Shimizu et al. concluded that PBL can be done effectively with Asian students; however, because they are accustomed to professors as authority figures, the role of PBL facilitators is critical in creating an environment that welcomes participation, cooperation, reflection, and constructive discussion (22). As students may come from diverse backgrounds and have different characteristics, including different countries and cultures of origin, personalities, and disabilities, it is important that they learn from each other by respecting and honoring their differences. With growing diversity among our students, our role as faculty members is to facilitate inclusive learning and value student diversity. To achieve this, one strategy was to incorporate time to talk about group rules at the beginning of the first PBL (23) and reflect on them each time they started a PBL class. In this way, students could set their own group rules and have opportunities to discuss them if the group experience was not going well. This strategy has been discussed in the literature. Faculty could also intervene as needed while encouraging students to resolve their concerns with each other.

Additionally, students who identified as males exhibited a greater increase in growth mindset scores than those who identified as females. One of the male stereotypes in our society is to avoid weakness (24); thus, admitting their own mistakes may be more challenging for males than females. Moreover, largely due to the majority of elementary school teachers being females, the feminization of education has been documented, systematically benefitting female learners rather than males (25). Thus, males are disadvantaged in their learning from the beginning of their formal educational journey. Considering both backgrounds, as PBL employs a non-traditional, active learning method and utilizes small-group learning, we believe that those who identified as males perceived that they were able to grow through this experience.

After examining the differential impacts of PBL on demographic groups, it is important to explore potential strategies to address and mitigate these disparities. Enhanced faculty training on cultural competency and diversity can be instrumental in fostering an inclusive learning environment that respects and capitalizes on the diversity of student backgrounds. Furthermore, modifying PBL curriculum to include case studies and problems from a variety of cultural and gender perspectives could help ensure all students find the material relatable and engaging. This approach not only aligns with the educational goals of promoting equity and inclusion but also prepares students to function effectively in a diverse global society. Implementing these strategies requires careful consideration and coordination with broader institutional policies aimed at diversity and inclusion.

### 5.1 Limitations

Although this study has many strengths, providing evidence for the utilization of PBL for an undergraduate public health course using longitudinal data, we acknowledge some limitations. First, as this study was done at a single site, a private university in the southeast US, findings should be generalized with caution, particularly at a different setting or geographic location. To address this limitation, future studies could be conducted at different higher education institutions with different student demographics. Additional demographic and background information, such as students’ high school district ranking and socioeconomic status, could be included to further describe the sample and potentially explore their associations with student learning. Second, although this study illustrated many statistically significant associations among different areas of learning as well as demographic characteristics, the study only shows association, not causation. Further studies with rigorous methods (e.g., quasi-experimental study with control groups) are needed to establish causality among the variables of interest. Third, the questions used in the study are not instruments whose psychometric properties can be tested. However, the university-level survey items were created by expert staff as a part of the university’s Quality Enhancement Plan before the authors engaged with PBL. The instructor created the course-specific questions to gain feedback on the PBL modules students completed, as it was the first time she fully implemented PBL for the entire course. Future studies should consider using scales in which psychometric properties have been tested and established. Fourth, the study addressed some demographic characteristics and examined differences in how they responded over time; however, group composition is not taken into account. Future studies can take into account how groups are composed (e.g., the proportion of males and females, as well as students with different races/ethnicities) and its impacts on learning outcomes. Fifth, there is a potential for burnout among students as they took several surveys over the course of the semester. However, it is also one of the strengths of the study that we were able to examine the change over the course of the semester. Although challenges exist in tracking students after the semester is over, future studies could assess students beyond the completion of the course to examine longer-term impacts of PBL.

### 5.2 Lessons learned

In an effort to promote broader adoption of PBL in the public health classroom, the following guidance is provided to support implementation efforts. Key logistical considerations include the appropriateness of PBL as a modality, selecting an effective format, group formation strategies, PBL design, instructor feedback, and student accountability. PBL can be time-intensive, which may prove challenging for introductory courses that provide a surface-level survey of a wide range of topics. While this paper demonstrates the feasibility of using PBL in such a course, the type of course (i.e., introductory or upper-level) and course content that needs to be imparted should be weighed when determining the appropriateness of PBL as a modality. Likewise, course format (e.g., in-person, remote, hybrid) may impact the effectiveness of the PBL experience as it relates to student engagement. While PBL can be effective across formats (26), student feedback in the current study reflected a preference for in-person PBL to facilitate more efficient collaboration. When considering group formation, group sizes of five to eight students have been recommended (27). Group assignments can be random, though this strategy should be weighed against the pros and cons of more intentional assignments that promote group diversity.

Additionally, forethought should be given to whether it is more advantageous for group members to continuously work together or to rotate between groups across multiple PBL modules. The current study demonstrated stronger group rapport and enhanced collaboration as benefits to preserving group composition across modules. In terms of PBL design, students in the present research highly valued the real-world problems selected for each module, including inviting practitioners as guest speakers. Likewise, clear instructions are crucial for successfully achieving PBL learning objectives and promoting engagement. Students appreciated having steady guidance throughout each PBL module, including setting ground rules and expectations for work inside and outside the classroom. To promote student learning, timely instructor feedback throughout PBL modules is important, especially for progressive-release PBL modules where students require input that informs their subsequent PBL work. Student learning and equitable division of group work can also be encouraged using grading schemes that allow for both group and individual accountability. In doing so, careful consideration should be given to the impact of low- and high-stakes grading opportunities on student motivation and engagement.

Successful implementation of PBL in undergraduate public health courses also requires institutional support, beginning with high-level university commitments and extending to classroom-specific resources. Institutional commitment would include policy support regarding faculty promotion, tenure, and retention considerations, recognizing the additional effort and time faculty invest in PBL. By providing resources and access to faculty development opportunities, institutions can significantly reduce the workload and time-intensive aspects of PBL, allowing faculty to focus more on pedagogical innovation and less on logistical challenges. Administrative support is critical for sustaining PBL initiatives and encouraging more faculty to adopt the pedagogy. This includes providing training, technological tools, and learning materials specifically designed for PBL.

Faculty development programs are another important part of institutional support for successful PBL. Educators need training programs that equip them with the skills necessary to design and implement effective PBL modules and a community of engaged faculty to foster ongoing collaboration, evaluation, and exchange of ideas. Initiatives such as the Faculty Learning Community Fellowship at our university serve as a model, offering opportunities for faculty to learn and share best practices in PBL. Faculty development programs directly address the challenges observed in enhancing communication and metacognition in students. For example, through these programs, faculty are trained to design PBL modules that specifically target and foster these skills, as evidenced by the significant improvements reported in our study’s results. By equipping faculty with targeted strategies and techniques, such as norming PBL, the development of effective problems, and framing and facilitating reflective discussions, these development programs ensure that educators are better prepared to guide students through the complexities of PBL, directly contributing to the observed successes in student learning outcomes. This approach in faculty training enhances the effectiveness of PBL in developing crucial skills among students and ensures that these educational gains are consistently achieved across diverse PBL applications. Alongside this, effective assessment tools and feedback mechanisms are needed to evaluate student growth and provide guidance for their learning progression in PBL activities.

At the classroom level, the physical setup should be conducive to PBL, with adaptable/modular layouts for small group discussions and interactions, building an atmosphere of collaboration and student engagement. Additionally, the effective management of PBL in larger classes can be significantly enhanced with the support of Teaching Assistants. They could assist in managing PBL logistics, facilitating group discussions, and providing immediate feedback, ensuring each student’s active participation.

Comprehensive institutional support transforms PBL from a demanding endeavor into a sustainable and rewarding teaching method, ensuring its long-term success and viability in public health education.

## Scope statement

The authors of this manuscript examined the impacts of an active learning method, problem-based learning, which was implemented in an undergraduate public health course. This manuscript is being submitted to *Frontiers in Public Health*, specifically in the “Public Health Education and Promotion” section. One of the topics of interest in this section is “Innovative teaching and learning in health education and promotion,” The authors believe that our manuscript fits perfectly in this priority topic area. The authors also believe that the manuscript will contribute to improve the education of future public health and healthcare professionals. We thank you for your careful consideration and review of our manuscript.

## Data availability statement

The raw data supporting the conclusions of this article will be made available by the authors, without undue reservation.

## Ethics statement

The studies involving humans were approved by University of Miami Institutional Review Board. The studies were conducted in accordance with the local legislation and institutional requirements. The ethics committee/institutional review board waived the requirement of written informed consent for participation from the participants or the participants’ legal guardians/next of kin because electronic survey was given to participants. This was educational research with minimum potential risk.

## Author contributions

YM: Conceptualization, Investigation, Methodology, Supervision, Writing – original draft, Writing – review & editing. AF: Formal analysis, Investigation, Methodology, Validation, Writing – original draft, Writing – review & editing. AP: Formal analysis, Methodology, Visualization, Writing – original draft, Writing – review & editing. AR: Conceptualization, Writing – original draft. LM: Formal analysis, Investigation, Writing – original draft. DV: Data curation, Investigation, Writing – original draft. YV: Data curation, Investigation, Writing – original draft.
